# Using geographical information systems mapping to identify areas presenting high risk for traumatic brain injury

**DOI:** 10.1186/1742-7622-8-7

**Published:** 2011-11-04

**Authors:** Angela Colantonio, Byron Moldofsky, Michael Escobar, Lee Vernich, Mary Chipman, Barry McLellan

**Affiliations:** 1 Saunderson Family Chair in Acquired Brain Injury Research, Toronto Rehabilitation Institute-UHN, University of Toronto, Toronto ON, Canada; 2 Department of Occupational Science & Occupational Therapy, University of Toronto, Toronto ON, Canada; 3 Dalla Lana School of Public Health, University of Toronto, Toronto ON, Canada; 4 Department of Geography, University of Toronto, Toronto ON, Canada; 5 Sunnybrook Health Sciences Centre, University of Toronto, Toronto ON, Canada

**Keywords:** traumatic brain injury, geographic information systems, geographic visualization, spatial analysis

## Abstract

**Background:**

The aim of this study is to show how geographical information systems (GIS) can be used to track and compare hospitalization rates for traumatic brain injury (TBI) over time and across a large geographical area using population based data.

**Results & Discussion:**

Data on TBI hospitalizations, and geographic and demographic variables, came from the Ontario Trauma Registry Minimum Data Set for the fiscal years 1993-1994 and 2001-2002. Various visualization techniques, exploratory data analysis and spatial analysis were employed to map and analyze these data. Both the raw and standardized rates by age/gender of the geographical unit were studied. Data analyses revealed persistent high rates of hospitalization for TBI resulting from any injury mechanism between two time periods in specific geographic locations.

**Conclusions:**

This study shows how geographic information systems can be successfully used to investigate hospitalizaton rates for traumatic brain injury using a range of tools and techniques; findings can be used for local planning of both injury prevention and post discharge services, including rehabilitation.

## Background

Geographic information systems (GIS) describe a group of software tools and methods that are used to integrate and evaluate data from a variety of sources with geographic location as the underlying framework for integration [1,2]. These data may be mapped for visualization purposes, and their locational relationships may also be analyzed using tools from the field of spatial statistics. GIS has been used by epidemiologists to investigate associations between environmental exposures to, and the spatial distribution of, infectious disease [3-6]. GIS research in health and healthcare has primarily relied on government supported databases [7-10] of vital statistics to visualize mortality and morbidity.

While most large-scale studies have focused on disease, a substantial amount of GIS and health-related research has also investigated incidence and mortality related to injury [11,12]. In particular, research has focused on injury resulting in pedestrian mortality among adults [13-15] and children [16,17]. These studies have primarily been conducted to identify at-risk intersections or neighbourhoods within an urban centre, or to compare the effects of urban design or intervention programs on pedestrian safety. Subsets of these studies have also linked individual data with contextual effects and have found that injuries are not random events occurring within a geographic area; rather, an increased risk of injury has been linked to factors such as regional population density, unemployment rate, and various indicators of socio-economic status [12,14,18,19].

One area of injury research that has received surprisingly little attention in the GIS literature has been traumatic brain injury (TBI). TBI is a leading cause of death and disability [20], predominantly affecting two age groups: adolescents and young adults, where a large percentage of injuries occur as a result of motor vehicle crashes; and persons over the age of 75, where most injuries occur from falls [21,22]. Since many of these injuries are preventable, and a high proportion of people sustain these types of injuries, TBI represents a major public health concern, particularly in terms of injury prevention. In addition, because TBI can result in long term disability, better information on geographic patterns can inform resource allocation for post-injury care, including rehabilitation. The Centers for Disease Control and Prevention (CDC) have maps available online for viewing TBI mortality rates at national and state levels [23]; some states have also generated TBI mortality rates by county. However, there are no published reports in the peer reviewed literature specifically on TBI incidence across large geographic regions, and none to date in Canada. In Canada, hospital administrative data are directly linked to public health insurance records; thus, the presence of publicly insured health care ensures the the availability of administrative data for an entire population in Canada, which includes a representative sample of hospitalizations for TBI.

One previous research effort, broadly related to our study, focused specifically on geographic disparity in all-cause premature mortality in Ontario [24]. Standardized mortality ratios (SMR) were used to identify geographic areas with higher mortality than expected at three population levels: regional, district health council, and public health unit. Results showed higher than expected mortality rates in some large regions, specifically in Northern Ontario, and that geographic disparities were clearly greater and more easily differentiated when analysed for smaller geographic areas. Altmayer et al. [24] also noted that such disparities reflect the underlying distribution of population health determinants.

The present study, therefore, is an exploratory analysis to establish whether GIS methods can be used for investigating regional differences in rates of hospitalizations for TBI from all mechanisms of injury in Ontario, Canada.

## Results & Discussion

### Introduction

Geographic location of hospitalizations for TBI, aggregated to regional counts by municipality, was examined for two separate periods eight years apart in the province of Ontario, Canada. Hospitalization rates for TBI were mapped by age and major mechanisms of injury (e.g., motor vehicle collisions or falls). A province-wide exploratory analysis was used to identify potential geographic areas of high risk. Further, mapping incidence at two different times aimed to show changes in rates over time, and to identify those areas with a persisting high risk for TBI occurrences. Although other studies have compared the incidence of TBI in urban and rural areas [18,25], this study collects and analyses within-province hospitalizations for TBI for each municipality.

This study was designed to determine the potential of GIS methods, focusing on data exploration and hypothesis-generation rather than on formal hypothesis-testing. As a result, the study went through two iterations of data preparation and analysis: first, to identify data characteristics, and to test software and the methodological approach; and second, to try to resolve some of the methodological issues identified in the first iteration and apply the most promising methods. A prior technical paper provides additional methodological details [26]. Since the purpose of this paper is to explain the methods via the example data set, the methods and results section of this paper are combined. This section contains a discussion of how and why different analytical methods are used. Also, it contains information on how to interpret the results for this data.

#### Data sources

The data on hospitalizations for TBI were obtained from the Ontario Trauma Registry's Minimum Data Set for two time periods, 1993-94 and 2001-02. These individual-level data were geographically located using the Ministry of Health "Residence Code", used by the ministry for service provision, and were based on the address recorded by the Ontario Health Insurance Plan - the province's public health insurance plan - for each patient. The population and socio-demographic data used were publicly available census information, collected and distributed by Statistics Canada ("StatCan"), for the census years 1991 and 2001. Through the Data Liberation Initiative of StatCan, the University of Toronto Library System licenses these data for research purposes. Geographic reference map files from StatCan's Census geography are also available through this program. Supplementary geographic data files from the library and other sources were also used for map creation and data analysis. Table 1 summarizes the two main data sources.

**Table 1:** Summary of two main data sources

Ontario Trauma Registry "Minimum Data Sets"	
Time periods	• Apr 1993 - March 1994 (n = 12,922)
	• Apr 2001- March 2002 (n = 10,782)

Criteria for inclusion	• Age at time of accident > 15 years
	• Acute hospital admission with ICD9 diagnosis codes 850-854 indicating traumatic brain injury

Variables	• Age at time of accident by 5 year cohort
	• Gender
	• Mechanism of injury (Motor vehicles, Falls, Other)
	• Geographic location (MOH Residence Code 1993 and 2001; usually corresponds to municipality; based on address of patient recorded by the Ontario Health Insurance Plan)

**Statistics Canada Census Geographic files**	

Time periods	• 1991 Census
	• 2001 Census

Geographic location:	• 1991: 951 CSDs
Census subdivision (CSD) - usually corresponds to municipality	• 2001: 586 CSDs (reduction due to municipal amalgamation)

Variables*	• Population counts
	• Age and gender by 5-year cohorts
	• Socio-economic indicators (including Occupation, Income and Education related indicators)

* data missing or suppressed for some CSDs

#### Uses of GIS to inform public health decision-making

**Figure 1:**
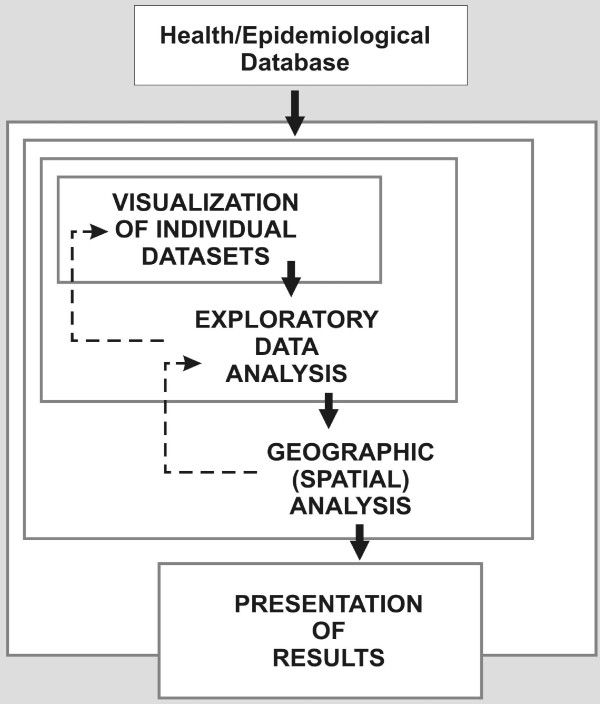
**Uses of GIS to inform public health decision-making**. Derived from the "exploratory spatial data analysis" process of Dragićević et al. [27]

For the purposes of this paper, the main uses of GIS can be categorized into four phases, as visualization, exploratory data analysis, geographic (spatial) analysis, and presentation of results (See Figure 1, derived from Dragićević et al [27]). The boundaries between these uses are sometimes blurred, and the distinctions between them may be somewhat semantic; while working in a GIS environment they occur more as way stations along a continuous process rather than as discrete steps. In fact, each successive use may be seen as an extension or enhancement of the previous one.

**Figure 2:**
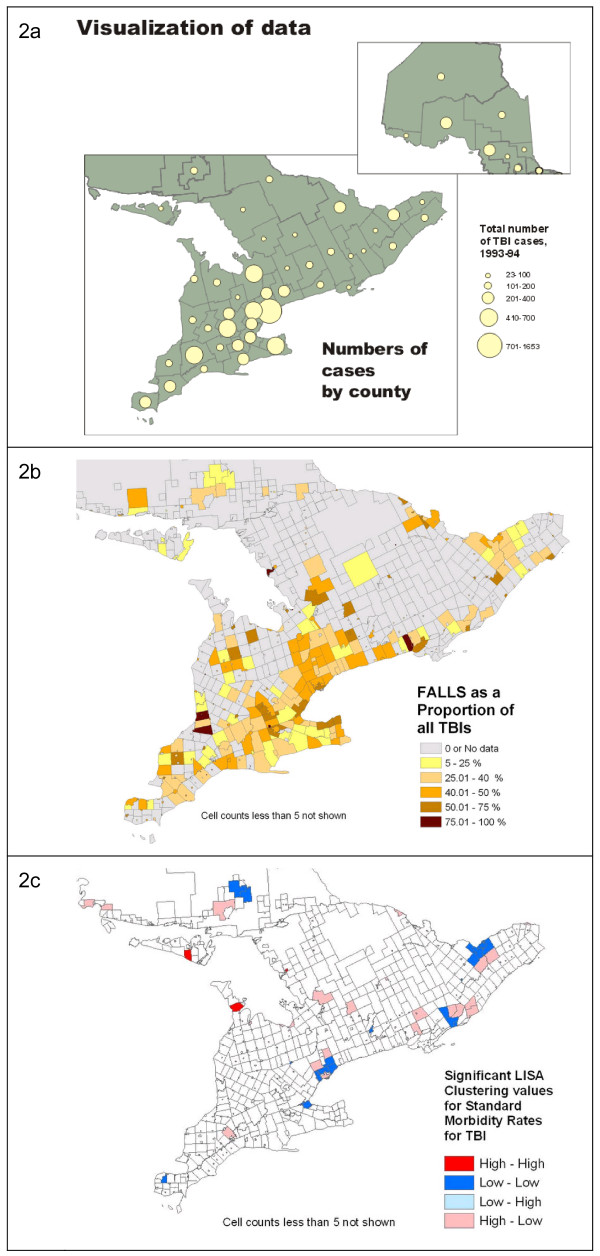
**Examples of maps showing visualization, exploratory data analysis, and geographic (spatial) analysis**. 2a: Example of visualization. Providing an overview and visual illustration of data sets, and putting them in geographic context, is an important function of GIS and mapping. Here we view an overall look at the distribution of cases of TBI from the 1993-94 data set. 2b: Example of exploratory data analysis. Maps of ratios or proportions of subsets of the participant population, by different variables, can be created for visual examination. The cartographic methods used for representing data have a significant impact on their visual interpretation. Many municipalities are small in area, and so are practically invisible in the choropleth map shown here. 2c: Example of geographic (spatial) analysis: Statistics such as the "Local Moran's I", generically referred to as the Local Index of Spatial Autocorrelation (LISA) can be used to identify areas of significant geographic clustering of data points, in this case, the initial calculation of standardized morbidity ratios by CSD for TBI.

The first phase, visualization, is defined herein as the act of representing a single data set on a map and examining it for patterns. In this study, counts of TBI hospitalizations were aggregated and mapped by geographical area, as shown in Figure 2a. Exploratory data analysis takes the visualization process further by comparing data sets by overlay of one time frame over another or calculation of statistics (Figure 2b) and includes tools ancillary to mapping, such as graphing or data brushing. The third phase, geographic (spatial) analysis, explicitly utilizes the methods of spatial statistics, which incorporate location and topological (i.e., neighbouring) relationships into the analysis of a dataset (Figure 2c). Finally, presentation of results represents the graphic communication of the results of analysis to an audience.

#### Methodological issues

In this study individual incident data were aggregated to regional counts; this means that the total number of injuries of each type and for each subpopulation were cross-tabulated by municipality. Using regional count data to analyze spatial clustering raises a number of issues and limitations related to the imposition of the "filter" of the aggregation units on the data [28]. Prime among these is the risk of ecological fallacy, or the geographical equivalent, the "modifiable areal unit problem [MAUP]". Simply put, this is the demonstrable risk that aggregation by different geographic "containers" will lead to variable results in statistical correlation [29]. Regional count analysis must "also balance the small-number problem with the spatial scale of the data" [28, pg. 201]. That is, small geographic units lead to small counts, which reduce the statistical stability of observed and estimated data. Therefore, deciding which geographic units to use is key.

**Figure 3:**
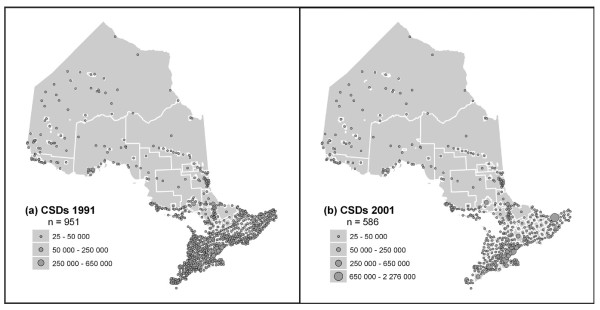
**Ontario Census subdivisions (CSDs) in 1991 (n = 951), and 2001 (n = 586)**.

In this study census subdivisions (CSDs) were used, as these were the smallest units of census geography that could be related to the incidence data available at the level of the municipality (Figure 3). It was necessary to use census units for the provision of demographic and socio-economic information that can be obtained; for example, age-cohort data were used to calculate SMR by CSD. Since another goal of the study was to compare rates from 1993-94 and 2001-02, using census geography also allowed comparable aggregation units to be created, and data compared, across time. Lastly, the potential for correlation of rates to socio-economic variables was also explored.

#### Data analysis

The first iteration of data exploration mapped and examined a wide variety of data comparisons, including comparisions of hospitalization rates to age distribution and mechanism of injury. Generally, expected patterns seemed to emerge: areas with older populations had higher rates, presumably due to falls; rural locations appeared to have higher rates than urban ones. We found that, although some intriguing patterns emerged, interpretation was limited by the methodological issues listed above (i.e., MAUP), as well as by some limitations in GIS functionality. Regarding the latter, the areas that needed to be improved were:

1. Ability to visualize and explore multivariate data relationships

2. Ability to control the method of creating neighbour relationships and other parameters for aggregation and spatial clustering analysis

3. Ability to compare patterns of spatial clustering over time

4. Ability to do regression analysis incorporating a spatial component.^1^

Therefore, in preparation for the second iteration of analysis, the methodological issues dealing with regional count data in this context, and limitations in GIS functionality, needed to be addressed.

The Local Indicator of Spatial Association (LISA statistic) was used [30,31] for analysis. This application identifies clusters of High-high CSDs (units of significantly high rates surrounded by other significantly high rates, after a randomization process and significance testing is applied), and High-low clusters (units of significantly high rates surrounded by significantly low ones.) The statistic also identifies significant clusters of low rates, but these were not considered in this study. Persistence of clusters between the two time periods studied was also examined. In addition to the LISA analysis, an alternative measure to identify clustering, the Getis-Ord Gi* statistic [32], was used to corroborate results.^2^

#### GIS methodological and functional issues

We dealt with the methodological issues in two ways. First, we decided to aggregate CSDs to achieve a "minimum population threshold," intended to stabilize rate calculations. This involves pooling together areas with small populations in order to provide enough of a population to determine rates. Second, use of an appropriate "rate-smoothing" method was made to overcome the problems of high rates based on low base populations [28,30]. The most common solution in the literature is the use of spatial empirical Bayes interpolation to smooth the data surface and eliminate "zero" values. Both of these operations required analysis of "nearest neighbours" for each geographic unit based on their relationship to surrounding units; to establish nearest neighbours, CSDs were aggregated and rates were smoothed. Notably, in this study, we decided that relationships should be based primarily on a network analysis, using transportation connections and distance to define nearest neighbours. This was also the basis for the definition of neighbours in the cluster analysis described below.

The limitations in GIS functionality were overcome by using two different GIS packages. Also, after the second iteration's visualization and data exploration stages, we decided to focus on points 2 and 3 above -- analysis of clustering of standardized rates of TBI hospitalization and comparison of spatial clustering patterns over time. This seemed to be the best way to identify areas with significant TBI occurrences.

#### Visualization and exploratory data analysis

Both the 1991 and 2001 maps of age-standardized TBI counts by municipality show a strong correlation to overall population distribution, as would be expected. (These maps are not illustrated here for confidentiality reasons.) Thus, more urbanized Southern Ontario shows the concentration of large counts. All other maps represent age-standardized TBI rates rather than counts, so differences in population sizes are no longer an issue.^3^

**Figure 4:**
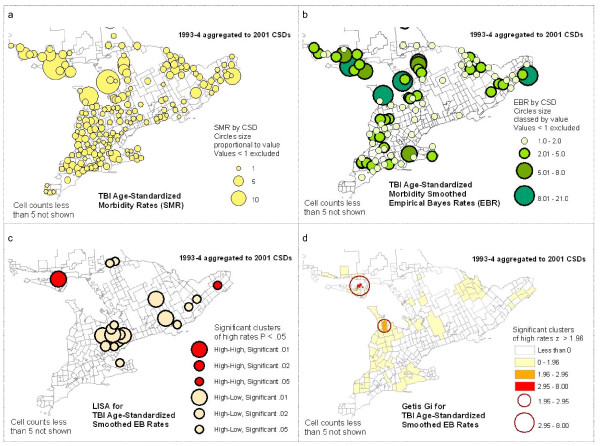
**Examples of mapping of TBI rates and cluster analyses**.

When SMR is mapped (see Figure 4a), the pattern is generally the inverse of population distribution, with rural areas and the North showing more high rates of hospitalizations, with a few outliers in the South. This pattern is maintained when empirical Bayes smoothed rates (EBR) are mapped (Figure 4b), and generally applies to both the 1993-94 and the 2001-02 data. Within this pattern, there are several areas where the higher values tend to cluster, or high outliers occur. Described generally, these are:

1. a large number of isolated communities in Northwestern Ontario

2. North Central Ontario (a collection of high rates)

3. on or near Manitoulin Island in Lake Huron (a large concentration of high rates)

4. spread across Southwestern Ontario (a collection of high rates)

5. scattered parts of South Central and Eastern Ontario (a few large outliers; these vary between time periods)

These findings highlight potential problem areas for further investigation. Our study follows up on this analysis using spatial analysis of clustering.

One aspect of this exploration, often neglected, is that the cartographic methods used for representing data have a significant impact on their visual interpretation. As an example, many municipalities are small in area and so are practically invisible in the shaded area (choropleth) maps generated by default in GIS statistical software. However, when data by CSD are mapped using circles proportional in size to the data, these data become more visible, and distinct clustering patterns may be better perceived (See Figure 4). To accomplish this, the default cartographic rendering in most GIS packages will need to be overridden with appropriate customized representation, or symbolization.

#### Spatial analysis of clustering - LISA and Getis-Ord Gi* statistics

The importance of geographic clustering of high hospitalization rates has not yet been established. Since TBI is not "contagious", the assumption is that an underlying phenomenon may exist which is related to proximity, connectivity, or other environmental contextual factors; this would influence high rates to be grouped together spatially. If this clustering is found, further investigation into these potential factors should be undertaken.

The LISA (Local Moran's i) and Getis-Ord Gi* methods for identifying clusters each take a slightly different approach to the task. In terms of practical interpretation, both methods identify significant High and Low clusters, i.e., High or Low geographic units neighbouring on similarly High or Low units, where units in this case are CSDs. The LISA also identifies anomalous clusters, i.e., High units surrounded by Low ones, or vice versa. In this study we are interested in clusters of High values only. For interpretation purposes, maps were constructed showing only significant High clusters as classed, colour-coded circles sized according to multiple significance levels (p < .01, p < .02, or p < .05) (See Figure 4c and 4d.) This provides a more nuanced tool for interpretation of results than a simple binary representation.

The results of the spatial analysis of clustering generally reinforce the visual analysis of the data exploration maps: many of the same groupings of high EBR values identified visually appeared as significant High-high clusters resulting from the LISA analysis, although at varied levels of significance. In contrast, many of the high outliers which were geographically isolated did not re-appear as significant clusters at all, either in the High-high or the High-low category. This is to be expected, as the relationships among neighbouring CSDs affects the cluster analysis; for example, a value can be high, but if surrounded by moderate values it will not be identified as a significant cluster. Comparing the results of the LISA clusters and the Getis-Ord Gi* clusters, most of the LISA High-high clusters are repeated as Gi* High values. There are some exceptions in both directions, but overall the two methods corroborate the clustering results.

#### Comparison of two time periods: 1993-94 and 2001-02

In order to make the mapping and analysis of the 1993-1994 and 2001-2002 TBI data compatible, we aggregated the data to common geographic units and merged the 2001 CSDs to match the more numerous 1991CSD boundaries as closely as possible. This created a "lowest common denominator" map of comparable geographic units. Comparison was done in two ways: a visual comparison of patterns between EBR and clustering maps for the two time periods, and an analysis of persistence of significantly high clusters between the two periods.

**Figure 5:**
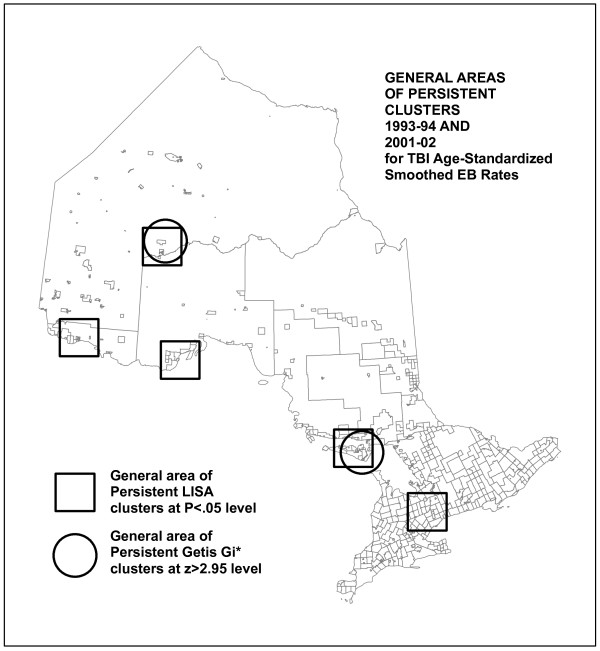
Persistent high clusters for 1993-94 and 2001-02 data, identified by LISA and Getis-Ord Gi* cluster analyses.

**Figure 6:**
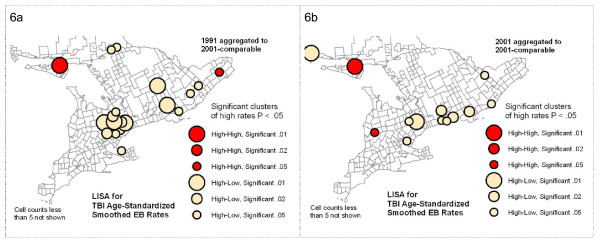
**LISA Cluster maps contrasting results using 1993-94 data with 2001-02 data, each aggregated to 2001-comparable CSDs**. High-low LISA clusters in Southern Ontario show similar patterns, but in different CSDs.

Visual comparison showed significant similarities in the patterns of high EBR values and clustering between the earlier and later data series. The most stable were the patterns noted above as points 1, 2 and 3, that is, high rates of TBI incidence in Northwestern and North Central Ontario, and around Manitoulin Island. The analysis of persistence of clustering, however, found a fairly small number of individual CSDs that are identified as clusters in both time periods (see Figure 5). This map specifically compares TBI smoothed EBR significant clustering statistics (LISA and Gi*) between two time periods, showing persistence of clusters by each method. For the LISA statistic, there are few persistent clusters: only nine keep the same classification from one time period to the next. A greater number of Gi* High clusters persist, but still a small proportion. This reflects the fact that even in the "stable" areas of high rates and clustering, on closer examination, there is some amount of shifting of high rates among neighbouring CSDs. Even where clusters do not persist, however, comparable patterns may be repeated. A good example of this is the High-low clusters identified by the LISA analysis in Southern Ontario (See Figure 6). These represent elevated rates with moderately low neighbours. That these exact clusters do not persist indicates they may be the result of a temporary situation or unique event. However, the fact that there is a similar pattern of other CSDs in the same general area with similar cluster characteristics may indicate that there is some mechanism at work that has a geographic component, or that similar conditions in these Southern Ontario communities result in similar kinds of TBI rate profiles, eight years apart.

#### Interpreting the results of the GIS analyses for this data

The persistence of high rates of TBI-related hospitalizations between the two time periods suggests the possibility of a chronic or recurring problem that may be the result of persistent risk factors. Moreover, similar geographic patterns of occurrence, even where exact locational persistence does not occur, may also signal a contextual element related to high incidence of TBI in which geographic location or contact between neighbouring populations plays a role. The rational follow-up to this analysis would be to focus on these identified areas of high rates (and of persistent clusters) for more a detailed study of demographics, mechanism(s) of injury and risk factors to see if these potential underlying operational factors can be discovered.

At this time, the accuracy of the patients' residence code has not been thoroughly assessed. In addition, examining changes in rates over time presented many challenges; this is because the underlying geographic groupings had changed such that smaller areas were merged into larger ones. This raises methodological issues that have been noted, such as the modifiable areal unit problem. It should also be noted that our study focused on hospitalization for TBI and as such represents the most severe of injuries. A much larger and more representative sample of TBI cases would have been available if data from all emergency rooms and acute care hospitalizations were included. However, emergency room cases that are not associated with a subsequent hospitalization are less likely to require post acute services such as in patient rehabilitation. We recognize, however, that even a "mild" TBI can have long term implications [33] and require subsequent care [34,35]. Thus, future analyses should be conducted on data that include all acute care, emergency room, and where possible physician visits.

In addition, mechanisms of injury for TBI vary by level of severity, where mechanisms such as being struck by an object may be more common with the inclusion of emergency room data [21]. Through ongoing research we plan to examine the counts by mechanism of injury. Preliminary data analyses show different geographic patterns by higher percentage of mechanism. For instance, TBI by falls have a higher concentration in a big city core whereas motor vehicle collisions are more likely to be in the suburban areas, presumably where there is a greater need for road travel.

It is also possible to compare the degree to which patients receive care in their own geographical area or go outside their regional funding units. In previous work, we have shown the percentage of persons who obtain care both within and outside their geographical area (local health integration network) [36]. This information can be used to direct funds towards unmet needs or to monitor financial impact of care in specific geographical areas. This concordance can also be mapped in future analyses; however, modeling shifts between hospitals would be extremely complex. Mapping according to patients' residence allows one to plan for home-based services for the long term and could thus greatly benefit patients since many persons with serious brain injury require long term support.

## Conclusion

This study demonstrates the value of exploratory data and spatial analysis using GIS for investigating hospitalization rates for TBI. This information can be used to guide local planning of both injury prevention strategies and services. The data can also be used by policy makers and advocates to justify additional resources for preventing and treating brain injury. Further, geographic analysis of hospitalization rates can be compared before and after specific interventions to determine impact. GIS in public health may be most effective when used by health professionals familiar with the conditions and concerns in the territory under examination for interactive mapping of different data sets and the development of hypotheses. In Ontario, funding has been decentralized to local health integration networks; public health units concerned with injury prevention also operate within certain geographical boundaries. Thus, a geographic level of analysis is particularly relevant. Our paper presents different types of data visualization that can highlight areas with high and low absolute numbers of hospitalizations for TBI, as well as information on high rates when base population rates are taken into account. Additionally, the predominance of different types of injury mechanisms can be examined. Geographic trends over time are also useful in order to determine whether patterns are stable or random. The outcomes from this study demonstrate the potential of this methodological approach and identify a number of areas for future investigation, such as uses of GIS for targeting rehabilitation and prevention services.

## Competing interests

The authors declare that they have no competing interests.

## Authors' contributions

All authors contributed to the conception and operationalization of this project. AC, as principal investigator, was involved in moving this manuscript into production and peer review, and contributed substantively to revisions of this manuscript; BMo was involved in preparation of the first draft of the manuscript; ME and LV assisted with analyses and provided methodological support. All authors read and approved the final manuscript.

## Endnotes

1. Since this study was done ArcGIS and some other GIS packages have begun to include functionality for points 3 and 4

2. The software used for the data exploration and the LISA analysis was GeoDa (Spatial Analysis Lab, Department of Geography, University of Illinois; now at GeoDa Center for Geospatial Analysis and Computation at Arizona State University - http://geodacenter.asu.edu/) [31]. For the network analysis, the Getis-Ord Gi* statistic, and for mapping purposes, ArcGIS 8.3 was used (http://www.esri.com.)

3. All the map examples in Figure 4 show the 1993-94 data aggregated to 2001 CSDs which were based on larger amalgamated municipalities; the 2001-02 data were similarly mapped.

## References

[B1] Robinson TP Spatial statistics and geographical information systems in epidemiology and public health Adv Parasitol 2000 47 81 128 10997205 10.1016/s0065-308x(00)47007-7

[B2] Kistemann T Dangendorf F Schweikart J New perspectives on the use of Geographical Information Systems (GIS) in environmental health sciences Int J Hyg Environ Health 2002 205 169 181 10.1078/1438-4639-00145 12040915

[B3] Nuckols JR Ward MH Jarup L Using geographic information systems for exposure assessment in environmental epidemiology studies Environ Health Perspect 2004 112 1007 1015 10.1289/ehp.6738 15198921 PMC1247194

[B4] Jarup L Health and environment information systems for exposure and disease mapping, and risk assessment Environ Health Perspect 2004 112 995 997 10.1289/ehp.6736 15198919 PMC1247192

[B5] McLafferty SL GIS and health care Annu Rev Public Health 2003 24 25 42 10.1146/annurev.publhealth.24.012902.141012 12668754

[B6] Cromley EK GIS and disease Annu Rev Public Health 2003 24 7 24 10.1146/annurev.publhealth.24.012902.141019 12668753

[B7] California Department of Pesticide Regulation Databases http://www.cdpr.ca.gov/dprdatabase.htm

[B8] Center for Health Applications of Aerospace Related Technologies http://geo.arc.nasa.gov/sge/health/chaart.html

[B9] World Health Organization European Health and Environment Information System for Disease and Exposure Mapping and Risk Assessment (EUROHEIS) 2005 Copenhagen

[B10] National Cancer Institute Cancer Mortality Maps & Graphs 2005 Bethesda, MD21986599

[B11] Aultman-Hall L Kaltenecker MG Toronto bicycle commuter safety rates Accid Anal Prev 1999 31 675 686 10.1016/S0001-4575(99)00028-7 10487343

[B12] Yiannakoulias N Rowe BH Svenson LW Schopflocher DP Kelly K Voaklander DC Zones of prevention: the geography of fall injuries in the elderly Soc Sci Med 2003 57 2065 2073 10.1016/S0277-9536(03)00081-9 14512238

[B13] Hijar M Trostle J Bronfman M Pedestrian injuries in Mexico: a multi-method approach Soc Sci Med 2003 57 2149 2159 10.1016/S0277-9536(03)00067-4 14512245

[B14] Lascala EA Gerber D Gruenewald PJ Demographic and environmental correlates of pedestrian injury collisions: a spatial analysis Accid Anal Prev 2000 32 651 658 10.1016/S0001-4575(99)00100-1 10908137

[B15] Wang S Smith PJ In quest of 'forgiving' environment: residential planning and pedestrian safety in Edmonton, Canada Plan Perspect 2001 12 225 250

[B16] Baker SP Waller A Langlois J Motor vehicle deaths in children: geographic variations Accid Anal Prev 1991 23 19 28 10.1016/0001-4575(91)90031-Y 2021400

[B17] Braddock M Lapidus G Cromley E Cromley R Burke G Banco L Using a geographic information system to understand child pedestrian injury Am J Public Health 1994 84 1158 1161 10.2105/AJPH.84.7.1158 8017545 PMC1614766

[B18] Gabella B Hoffman RE Marine WW Stallones L Urban and rural traumatic brain injuries in Colorado Ann Epidemiol 1997 7 207 212 10.1016/S1047-2797(96)00150-0 9141644

[B19] Cusimano MD Chipman M Glazier RH Rinner C Marshall SP Geomatics in injury prevention: the science, the potential and the limitations Inj Prev 2007 13 51 56 10.1136/ip.2006.012468 17296690 PMC2610555

[B20] Corrigan JD Selassie AW Orman JA (Langlois) The epidemiology of traumatic brain injury J Head Trauma Rehabil 2010 25 72 80 10.1097/HTR.0b013e3181ccc8b4 20234226

[B21] Colantonio A Saverino C Zagorski B Swaine B Lewko J Jaglal S Vernich L Hospitalizations and emergency department visits for TBI in Ontario Can J Neurol Sci 2010 37 783 790 21059539 10.1017/s0317167100051441

[B22] Das-Gupta R Turner-Stokes L Traumatic brain injury Disabil Rehabil 2002 24 654 665 10.1080/09638280110109282 12296981

[B23] Centers for Disease Control and Prevention Injury Maps 2005 Atlanta

[B24] Altmayer CA Hutchison BG Torrance-Rynard VL Hurley J Birch S Eyles JD Geographic disparity in premature mortality in Ontario, 1992-1996 Int J Health Geogr 2003 2 7 10.1186/1476-072X-2-7 14561226 PMC222916

[B25] Woodward A Dorsch MM Simpson D Head injuries in country and city. A study of hospital separations in South Australia Med J Aust 1984 141 13 17 6738403 10.5694/j.1326-5377.1984.tb132660.x

[B26] Moldofsky B Ngan J Colantonio A Methods developed for using geographical information systems to inform targeted rehabilitation and prevention services for traumatic brain injury: analysis of regional count data at the census subdivision level GCUT - GIS and Cartography Technical Paper Series 2008 University of Toronto http://www.geog.utoronto.ca/about/publications/gcut/gcut_home

[B27] Dragićević S Schuurman N Fitzgerald JM The utility of exploratory spatial data analysis in the study of tuberculosis incidences in an urban Canadian population Cartographica 2004 39 2 29 39 10.3138/6338-8M7X-4H12-30T9

[B28] Waller LA Gotway CA Applied Spatial Statistics for Public Health Data 2004 Atlanta: John Wiley & Sons

[B29] Gotway-Crawford C Young LJ King G, Rosen O, Tanner MA A spatial view of the Ecological Inference Problem Ecological Inference: New Methodological Strategies 2004 New York: Cambridge University Press

[B30] Anselin L Syabri I Kho Y GeoDa: An introduction to spatial data analysis Geogr Anal 2006 38 5 23 10.1111/j.0016-7363.2005.00671.x

[B31] Anselin L Local indicators of spatial association (LISA) Geogr Anal 1995 27 93 115

[B32] Getis A Ord JK Longley P, Batty M Local spatial statistics: an overview Spatial Analysis: Modeling in a GIS Environment 1996 Cambridge: Wiley 261 267

[B33] O'Connor C Colantonio A Polatajko H Long term symptoms and activity limitations after traumatic brain injury: a ten year follow-up Psychol Rep 2005 97 169 179 16279322 10.2466/pr0.97.1.169-179

[B34] Gagnon I Galli C Friedman D Grilli L Iverson G Active rehabilitation for children who are slow to recover following sport-related concussion Brain Inj 2009 23 12 956 964 10.3109/02699050903373477 19831492

[B35] Wei W Liu ML Fergenbaum J Comper P Colantonio A Work-related mild to moderate traumatic brain injuries due to falls Brain Inj 2010 24 11 1358 1363 10.3109/02699052.2010.506635 20715899

[B36] Colantonio A Vander Laan R Parsons D Zagorski B Mohan M Local Health Integration Network specific Acquired Brain Injury Reports http://www.onf.org/index_abi.html

